# Isolation and Genetic Characterization of Three Palyam Serogroup Orbiviruses From *Culicoides* Spp. and Associated Infections in Cattle in Yunnan, China

**DOI:** 10.1155/tbed/6615175

**Published:** 2025-08-25

**Authors:** Zhenxing Yang, Yuwen He, Susheng Li, Jinxin Meng, Nan Li, Jinglin Wang

**Affiliations:** ^1^Yunnan Tropical and Subtropical Animal Viral Disease Laboratory, Yunnan Animal Science and Veterinary Institute, Kunming 650224, China; ^2^Yunnan Provincial Key Laboratory of Public Health and Biosafety, Kunming Medical University, Kunming 650500, China

## Abstract

Palyam serogroup virus (PALV) is a potential pathogen prevalent in Asia, Australia, and Africa that may cause abortion and teratogenesis in ruminants, especially cattle. In this study, we isolated three PALV strains (MY_C17, ML_C40, and SZ_C06) from *Culicoides* spp. in Yunnan, China, and obtained their complete genome sequences via next-generation sequencing. The phylogenetic analysis of Seg-5, Seg-7, and Seg-9 indicated that PALVs can be grouped based on their geographical origins: Asia, Australia, and Africa. The three isolates in this study belong to the Asia group. However, the Central African Republic strain (AR_B_2032) and the India strain (DVTD) form a separate branch, provisionally named the CI group. In the VP2 phylogenetic tree, all PALV strains can be grouped into six significant branches, designated as A through F. The three isolates are clustered in Groups A, E, and F and are shown to belong to the D'Aguilar virus (DAGV), Chuzan virus (CHUV), and Bunyip Creek virus (BCV) serotypes, respectively. Seroepidemiological surveys were conducted on local cattle using neutralization tests, which revealed seroprevalence rates of neutralizing antibodies against BVC, DAGV, and CHUV in Yunnan cattle serum of 3.4% (12/350), 6.9% (24/350), and 16.9% (59/350), respectively. This marks the first-ever isolation of CHUV, specifically BCV and DAGV, from vector *Culicoides* spp. in Southwest China. It was also proved that in this region, at least three serotypes of PALV circulated among *Culicoides* spp. in nature and infected domestic cattle.

## 1. Introduction

Palyam serogroup virus (PALV) is an orbivirus within the family Reoviridae that is predominantly found in Africa, Asia, and Australia and is primarily spread by mosquitoes, midges, and ticks [1–3]. To date, at least 13 different viruses of this serogroup have been grouped into 10 antigenic complexes or serotypes: Chuzan, Kasba, Vellore, Abadina, Marrakai, Petevo, Bunyip Creek, Marondera, Apies River, D'Aguilar, CSIRO Village, and Gweru [4, 5]. Kasba virus and Chuzan virus (CHUV) belong to the same serotype and are more commonly known as CHUV in Asia.

CHUV, one of the most representative serotypes of PALV, was first isolated in 1985 from the blood of calves and *Culicoides* (oxystoma) in Japan and was believed to be a causative agent of bovine congenital disease characterized by hydranencephaly and cerebellar hypoplasia (HCH) [6, 7]. Subsequently, by inoculating the brains of suckling mice with CHUV, Yamaguchi et al. [8, 9] showed that the virus has an affinity for immature nerve cells in the brains of mice and can cause primary encephalomalacia lesions similar to those seen in calves with HCH syndrome. Additional serotypes of PALV appear to be involved in the occurrence of abortions and congenital malformations in cattle; however, the pathogenic importance of most of these serotypes is still unknown [10].

Like other members of the *Orbivirus* genus, the PALV genome is composed of 10 dsRNA segments, labeled Seg-1 to Seg-10, that encode seven structural proteins (VP1–VP7) and four nonstructural proteins (NS1, NS2, NS3, and NS3a) [11]. These structural proteins form the virion's double-layered capsid. The inner layer (core) is composed of two major proteins (VP3 and VP7) and three minor proteins (VP1, VP4, and VP6), and the outer layer is composed of proteins VP2 and VP5 [12]. The VP2 protein encoded by Seg-2 is a major neutralizing antigen that features a serotype-specific determinant and exhibits the most significant variation in its genome sequence [13]. The NS1, VP7, and VP9, encoded by Seg-5, Seg-7, and Seg-9, respectively, are highly conserved across all PALVs. They display genomic sequence variations that correspond to the geographic origins of the virus isolates, regardless of their serotype [12, 13].

So far, three serotypes (Chuzan, Bunyip Creek, and D'Aguilar) have been isolated from Asia, six (Abadina, Apies River, CSIRO Village, D'Aguilar, Petevo, and Marondera) from Africa, and four (Bunyip Creek, CSIRO Village, D'Aguilar, and Marrakai) from Australia [14, 15]. In China, CHUV was first isolated in 2012 from the blood of sentinel cattle in Yunnan Province [16] and later from sentinel cattle in the Guangxi Zhuang Autonomous Region [17]. In 2016–2017, Wang et al. [18] detected CHUV positivity in the blood of domestic yaks collected from the Qinghai–Tibet Plateau and successfully isolated the virus. However, there have been no reports of the isolation of other PALV serotypes other than CHUV in China.

During an arbovirus survey in Yunnan Province conducted between 2022 and 2023, three new PALV isolates were obtained from *Culicoides* spp. collected from Jinghong, Mengla, and Shizong counties, belonging to three serotypes: Chuzan, Bunyip Creek, and D'Aguilar. The present study describes the isolation and molecular characteristics of these viruses, along with their phylogenetic relationships to members of PALV. In addition, we investigated the prevalence of PALV in local cattle using serum neutralization tests (SNTs).

## 2. Materials and Methods

### 2.1. Sample Collection

#### 2.1.1. Culicoides Capture

**Figure 1:**
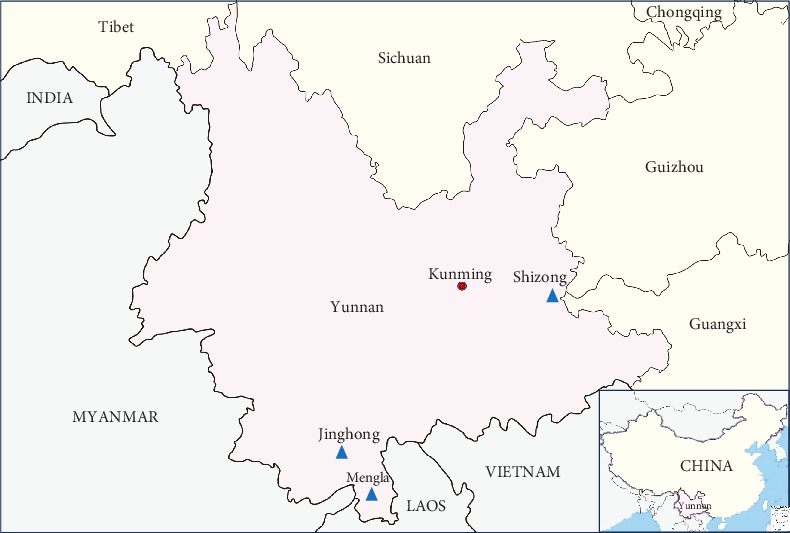
Geographic location of the three sampling sites in Yunnan province, China. (The map is drawn to a proportional scale of 1:6,200,000.) Mengla, Jinghong, and Shizong are indicated by a blue triangle.

From 2022–2023, midge specimens were collected overnight using light traps in cowsheds located in the rural areas of Jinghong, Mengla, and Shizong counties in Yunnan Province (Figure 1). After refrigerating, the collection bag at −20°C for 15 min, the insects were removed, and the *Culicoides* spp. were quickly selected using a stereomicroscope. Every 100 *Culicoides* spp. collected at the same time and location were grouped into one cryopreservation tube and rapidly frozen in a liquid nitrogen tank for preservation before being transported to the laboratory for viral isolation.

#### 2.1.2. Serum Sample Collection

In August 2022, 350 serum samples were collected from domestic cattle aged at least 1 year in Jinghong, Mengla, and Shizong counties, which are also districts where midge specimens are collected. The collected sera were transported to the laboratory and stored at −80°C for future examination. So far, no vaccine has been used in China, and the cattle were not previously immunized with the PALV vaccine.

### 2.2. Cell Cultures and Viral Isolation

As described previously, *Aedes albopictus* (C6/36) and baby hamster kidney (BHK-21) cells were used for viral isolation [19, 20]. Each grouping of 100 *Culicoides* spp. was homogenized in MEM using a Tissuelyser-600 (Jingxin Company, Shanghai, China). The homogenates were centrifuged, and the supernatant was then inoculated onto monolayers of C6/36 and BHK-21 cells in a cell culture plate for 7 days. The cells were monitored daily for cytopathic effects (CPEs) over three consecutive passages, and the supernatants from cultures that exhibited CPEs were collected and stored at −80°C for further analysis.

### 2.3. Initial Identification of the Virus

Following the manufacturer's guidelines, the supernatant from cell cultures displaying CPEs was processed for viral nucleic acid extraction using a TIANamp Virus RNA kit (Tiangen Biotech Company, Beijing, China). As previously reported, the extracted nucleic acids were detected via RT-qPCR using primers and probes specific to bluetongue virus (BTV), Akabane virus (AKAV), Tibet orbivirus (TIBOV), Banna virus (BAV), epizootic hemorrhagic disease virus (EHDV), and PALV, respectively [21, 22].

### 2.4. Amplifying Viral Genome and NGS Sequencing

#### 2.4.1. Amplifying Viral Genome

Viral dsRNA was reverse-transcribed into complementary DNA (cDNA) using the full-length amplification of cDNA (FLAC) technique, as previously described [23, 24]. In brief, the 3′ ends of viral dsRNA were ligated to an anchor primer and purified using a Monarch PCR & DNA Cleanup kit (NEB, USA). Then, cDNA was synthesized using the PrimeScriptTM II Reverse Transcriptase kit (Takara, Dalian, China). The resulting cDNAs were amplified using the primer 5-15-1 [23] by PrimeSTAR GXL DNA Polymerase (TaKaRa, Dalian, China). The above methods were carried out according to the kit manual.

#### 2.4.2. NGS Sequencing

The amplified products were analyzed in 1% agarose gel (90 V for 2–3 h) and then sent to Magigen Company (Guangzhou, China) for NGS sequencing. After preparing the reads, checking the sequences, and filtering the data, de novo assembly was performed using Abyss (v2.0.2) [25] and SOAPdenovo (v2.04) [26] to construct sequences for each genomic segment of these isolates. These sequences were then further verified with PCR using multiple primers.

### 2.5. Sequence Analysis and Phylogenetic Constructions

Reference sequences of PALVs together with an out-group (BTV) were obtained from GenBank on 1 September, 2024, and are presented in Supporting Information 1: Table S1. Open reading frames (ORFs) of viral genomic segments were identified and translated into amino acid (aa) sequences using DNAstar Lasergene EditSeq (v7.1.0). Multiple alignments of consensus sequences were performed using the CLUSTAL W program, and nucleotide (nt) and aa identities were calculated using BioAider (V1.627) [27]. Pairwise distance calculations and phylogenetic tree constructions were performed using MEGA-11 software with the neighbor-joining (NJ) statistical method, pairwise deletion, and a *p*-distance algorithm, and tested by bootstrapping 1000 replicates.

### 2.6. Serological Analyses

A SNT was used to detect neutralizing antibodies against the isolated viruses in the serum samples. First, to determine the TCID_50_/50 μL of each isolate, we performed tenfold dilutions of the supernatant from the isolates on BHK-21 cells using 96-well flat-bottom microtiter plates. After heat inactivation, the serum samples were diluted (1:10–1:1280) in a tissue culture medium without FBS. Each serum dilution is combined with an equal volume of virus containing 100 TCID_50_/50 μL and then, incubated at 37°C for 1 h with 5% CO_2_. Subsequently, 100 μL of a BHK-21 suspension (1 × 10^5^/mL) was added to each well and then incubated for 5–7 days at 37°C with 5% CO_2_. Each well was evaluated for the presence of CPEs. The neutralization titer was determined by the serum dilution that achieved the 50% neutralization endpoint and was assessed using the Spearman–Kärber method and expressed as the log^10^ reciprocal of the highest positive plasma dilution.

## 3. Results

### 3.1. Virus Isolation and Identification

A total of 10,800 *Culicoides* spp. specimens captured between 2022 and 2023 from Jinghong, Mengla, and Shizhong counties in Yunnan, China, were divided into 108 pools for virus isolation. Three isolates (MY_C17, ML_C40, and SZ_C06) were found to cause strong CPEs in the second-blind passage of BHK-21 cells after 72 h. The characteristics of the CPEs included cell rounding, lysis, and shedding. These isolates were then identified using RT-qPCR to detect the viral nucleic acids of AKAV, EHDV, BAV, BTV, PALV, and TIBOV. As a result, all three isolates were identified as PALV with Ct values less than 20. NGS-sequenced DNA samples of these strains produced via FLAC and the associated GenBank access numbers are provided in Table 1.

### 3.2. Genome Characteristics of the Three Strains

The sequence details for each segment of these isolates are presented in Supporting Information 2: Table S2. The genome lengths of ML_C40, MY_C17, and SZ_C06 are 18,914, 18,881, and 18,923 bp, with G + C contents of 39.31%, 38.85%, and 39.31%, respectively. All segment lengths of the three strains are highly conserved, except for the length of Seg-2, which is 3055, 3022, and 3064 bp and encodes 1002, 922, and 1006 aa residues, respectively. The analysis of the 5′ and 3′ noncoding regions (NCRs) revealed that each segment of the three strains shares highly conserved terminal nt sequences at the 5′ and 3′ NCRs (5′-GUUAAA and CUUAC-3′, respectively), except for Seg-9, which is CAUAC at the 3′ NCRs. Like most orbiviruses, the 5′ NCRs are shorter than the 3′ NCRs, except for Seg-1, which has longer 5′ NCRs (22 bp) than 3′ NCRs (20 bp). The 5′ and 3′ NCRs of the three strains comprised 3.48%, 3.51%, and 3.55% of their total genome, respectively (Supporting Information 2: Tables S2).

### 3.3. Sequence and Phylogenetic Analysis of Seg-5/NS1

We performed a sequence analysis of the NS1 gene from the three strains and other isolates from various locations. ML_C40, MY_C17, and SZ_C06 are closely related to the other PALVs, showing 81.3%–99.5%/92.5%–99.6% (nt/aa) sequence identity for NS1 (Supporting Information 3: Table S3). However, they are more distantly related to other orbiviruses, with nt/aa sequence identities less than 46.5%/32.6% for NS1 (data not shown).

**Figure 2:**
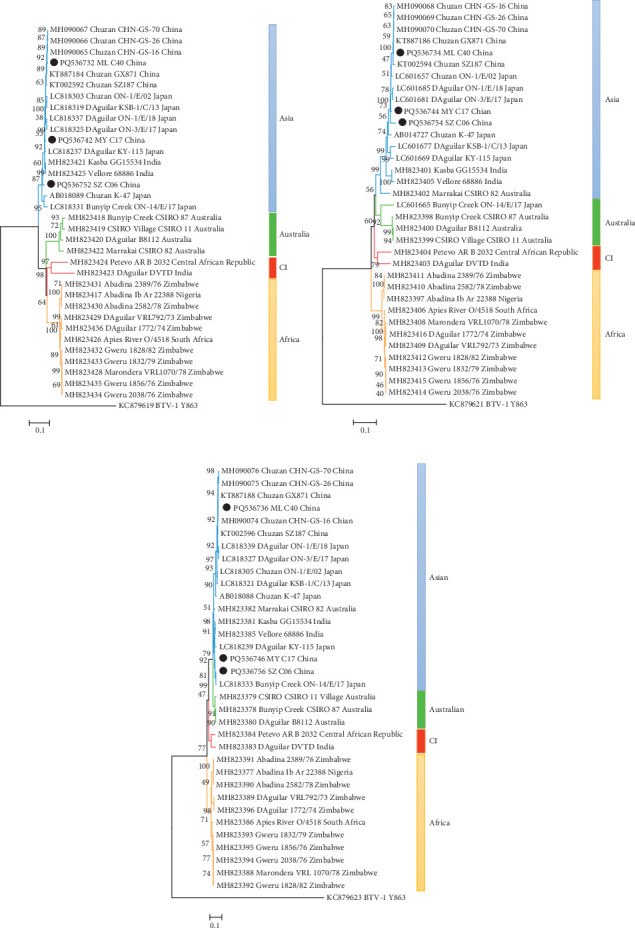
Phylogenetic analysis based on genome coding sequences of NS1 (A), VP7 (B), and VP6 (C) of three strains (ML_C40, MY_C17, and SZ_C06) with reference strains of recognized PALV. Each reference PALV strain is denoted as “GenBank accession number_ Serotype_ Strains number_ Country.” Outgroup viruses are denoted as “GenBank accession number_ Virus name_ Strains number.” The isolates in this study are depicted by black dots.

A phylogenetic tree was constructed by comparing the NS1 sequences of the three strains with those of other PALVs (Figure 2A). PALVs can be categorized into three branches based on their geographical origin: Asia, Australia, or Africa. However, the ARB2032 strain from the Central African Republic and the DVID strain from India form another independent group, provisionally named the CI group. The three strains isolated in this study were categorized as belonging to the Asia group. When all of the PALVs analyzed are compared, the overall level of NS1 nt and aa sequence identity is ≥80.9% and 91.9%, respectively (*μ* = 89.5%/96.5%; Table 2). When the Asian, Australian, and African isolates are considered as four separate branches, the sequence identity increases to ≥94.3%/96.8% (*μ* = 97.5%/98.6%) in the Asia group, ≥94.9%/97.7% (*μ* = 96.1%/98.5%) in the Australia group, ≥97.4%/98.1% (*μ* = 98.5%/99.5%) in the Africa group, and ≥83.5%/93.5% in the CI group. These results suggest that there are closer genetic relationships among isolates from the same geographic area.

### 3.4. Sequence and Phylogenetic Analysis of Seg-7/VP7

An analysis of the nt/aa sequence of the VP7 revealed 85.3%–99.8%/98.6%–100% sequence identity between the three strains and other PALVs (Supporting Information 4: Table S4). Their na/aa sequence of VP7 has less than 58.2%/53.1% identity compared to other orbiviruses (data not shown).

In the VP7 phylogenetic tree, PALVs can be regionally classified into four branches (Asia, Australia, Africa, and CI). The three strains were categorized as belonging to the Asia group (Figure 2B). Notably, in this phylogenetic tree, the CSIRO 82 strain, isolated from Australia, was grouped with the other strains in the Asia group. In contrast, the ON-14/E/17 strain, isolated from Japan, was grouped with the Australia group. Like the NS1 genome, VP7′s overall percentage identity at the nt level is very high when different groups are considered separately. The Asia, Australia, Africa, and CI groups show ≥90.3% (*μ* = 95.7%), 89.7% (*μ* = 94.6%), 96.9% (*μ* = 98.4%), and 89.5% identity, respectively. When all PALVs are considered together, this percentage decreases to ≥84.3% (*μ* = 89.6%; Table 2). The VP7 protein is still highly conserved, showing ≥97.7% (*μ* = 99.3%) identity at the aa level, which is significantly higher than the ≥84.3% (*μ* = 89.6%) observed at the nt level.

### 3.5. Sequence and Phylogenetic Analysis of Seg-9/VP6

An analysis of the nt/aa sequence of VP6 revealed 86.8%–99.8%/85.4%–100% sequence identity between the three strains and other PALVs (Supporting Information 5: Table S5). The na/aa sequence of VP6 has less than 38.1%/26.8% identity compared to other orbiviruses (data not shown).

Like the phylogenetic trees of NS1 and VP7, the VP6 phylogenetic tree can be categorized into four branches: Asia, Australia, Africa, and CI, and the three isolates in this study are clustered in the Asia group (Figure 2C). However, a strain isolated from Australia (CSIRO 82) was incorporated into the Asia group. The nt/aa sequences percentage identity of VP6 is ≥86.4%/84.5% (*μ* = 91.7%/91.0%) when all PALVs are considered together (Table 2). This percentage increases when examining different topotypes individually: ≥93.0%/91.9% (*μ* = 96.5%/96.4%) identity in the Asia group, ≥97.9%/97.4% (*μ* = 98.3%/98.0%) identity in the Australia group, ≥95.3%/93.7% (*μ* = 97.0%/96.3%) identity in the Africa group, and ≥93.2%/92.2% identity in the CI group. Unlike NS1 and VP7, which exhibit large numbers of synonymous mutations, VP6 demonstrates slightly lower identity at the aa level (≥84.5%) than at the nt level (≥86.4%), indicating the presence of nonsynonymous mutations.

### 3.6. Sequence and Phylogenetic Analysis of Seg-2/VP2

**Figure 3:**
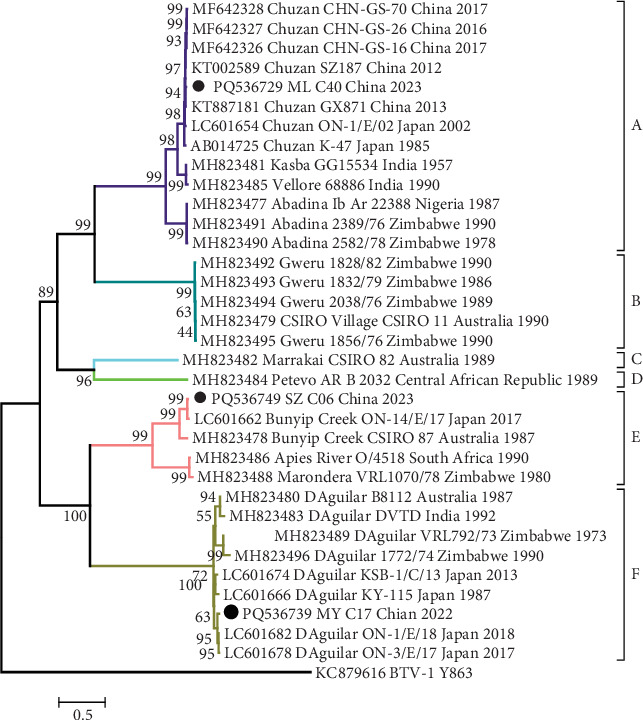
Phylogenetic analysis based on genome coding sequences of VP2 of three strains (ML_C40, MY_C17, and SZ_C06) with reference strains of recognized PALV serotypes. Each reference PALV strain is denoted as “GenBank accession number_ Serotype_ Strains number_ Country_ year of isolation.” Outgroup viruses are denoted as “GenBank accession number_ Virus name_ Strains number.” The isolates in this study are depicted by black dots.

Seg-2 (VP2) of PALV exhibits the highest levels of variation in genome sequence identity and size, correlating with the specificity of virus serotypes [13]. The analysis of percentage sequence identity highlights that variation is a critical characteristic of Seg-2/VP2, with an identity of ≥46.8% (*μ* = 60.7%)/35.4% (*μ* = 54.4%) at the nt and aa levels, respectively, across all PALVs included in the study (Supporting Information 6: Table S6). The VP2 of ML_C40 shared very high (97.6%–99.3%/98.0%–99.4%) nt/aa sequence identity with Chuzan strains, Z_C06 shared 91.0%–98.7%/94.9%–98.7% nt/aa sequence identity with Bunyip Creek, and MY_C17 shared 89.7%–98.2%/91.1%–99.4% with D'Aguilar strains. A phylogenetic analysis based on VP2 revealed that the ML_C40, SZ_C06, and MY_C17 strains are clustered in a clade with the Chuzan, Bunyip Creek, and D'Aguilar strains, respectively (Figure 3). These results indicate that MY_C17 is classified under the D'Aguilar serotype, ML_C40 is associated with the Chuzan serotype, and SZ_C06 is categorized as belonging to the Bunyip Creek serotype.

In the phylogenetic tree constructed using the nt sequences of VP2, all PALV types are organized into six main evolutionary branches: Groups A to F (Figure 3). These groups do not represent regional groupings, as shown in the NS1, VP7, and VP6 proteins, but rather seem to correlate with the virus's serotype properties. Four serotypes of PALV—Chuzan, Kasba, Vellore, and Abadina—constitute Group A and exhibit ≥79.4%/85.0% nt/aa sequence identity (Table 3). Gweru and CSIRO serotypes form Group B and share ≥99.4%/99.4% nt/aa sequence identity. Serotypes Marrakai and Petevo each form a group (Group C and Group D). Three serotypes, Bunyip Creek, Apies River, and Marondera, formed Group E, showing ≥70.9%/73.9% nt/aa sequence identity. Group F contains only one serotype (D'Aguilar) that exhibits nt/aa sequence identity ≥86.9%/88.5%. Based on these data, the Bunyip Creek, Apies River, and Marondera serotypes are the most divergent within Group E. In addition, the identity of the nt/aa sequence between different groups decreased significantly at ≤58.1%/53.1%.

### 3.7. Survey of Serum Antibodies in Local Livestock

We used the SNT method to detect antibodies against CHUV, Bunyip Creek virus (BCV), and D'Aguilar virus (DAGV), considering a titer of 1:16 or greater as indicative of a positive result. The positive rates of PALV antibody of cattle in Jinghong, Mengla, and Shizong counties were 28.6% (32/112), 27.4% (29/106), and 25.8% (34/132), respectively, and the overall positive rate was calculated at 27.1% (95/350; Table 4). The prevalence patterns differed for the three serotypes: CHUV was more common in the three locations, with the highest positive rate of 16.9% (59/350). In contrast, the remaining two serotypes (BAV and DAGV) had positive rates of only 3.4% (12/350) and 6.9% (24/350), respectively; however, the positive rate for DAGV is slightly higher than that of BAV.

## 4. Discussion

*Culicoides* biting midges are commonly found in regions ranging from temperate to tropical climates and are recognized or suspected of transmitting over 50 arboviruses that are significant for both veterinary and public health [28]. Some of these viruses are associated with diseases of such international importance that the Office International des Epizooties (OIE) has designated them as List A [29]. *Culicoides* spp. are also widespread in Yunnan, China, and in a previous arbovirus survey project, we detected or isolated BTV [30], Oya virus [31], EHDV [32], BAV [33], and TIBOV [34] from these insects. In the study, three viruses were isolated from *Culicoides* spp. captured from Mengla, Shizong, and Jinghong counties in Yunnan Province, China. They were tentatively identified as PALV through RT-qPCR, and their complete genomes were subsequently sequenced. Our present study provides comprehensive information on the genomic characterization, phylogenetic analysis, and identification of the serotypes of these viruses and discusses the serological investigation of viral antibodies at the site of viral isolation.

The NS1, VP7, and VP6 gene sequences are highly conserved among all PALVs and are often used as target genes for serogroup-specific detection [12]. Compared with other PALV isolates, the NS1, VP7, and VP6 of ML_C40, MY_C17, and SZ_C06 are closely related to those of different PALVs, exhibiting nt/aa sequence identities of 81.3%–99.8%/92.5%–100% for NS1, 85.3%–99.8%/98.6%–100% for VP7, and 86.8%–99.8%/85.4%–100% for VP6. However, they are more distantly related to other orbiviruses, with nt/aa sequence identities less than 46.5%/32.6% (NS1), 58.2%/53.1% (VP7), and 38.1%/26.8% (VP6), respectively. These results demonstrate that all three viruses belong to the PALV group, aligning with the identification results obtained through RT-qPCR.

The NS1 protein, encoded by the Seg-5, forms virus-specific tubular structures typically produced during orbivirus replication in infected cells [35]. NS1 is highly conserved and the percentage of nt/aa identity is ≥80.9% (*μ* = 89.5%)/≥91.9% (*μ* = 96.5%) when all strains are compared. This indicates that the intracellular tubules formed by NS1 are so essential to the virus that most changes in the nt sequence are synonymous mutations. The NS1 aa sequences of BTV and EHDV each contain 16 conserved cysteines, especially two cysteine residues at positions 336 and 339, which are considered crucial for correct protein formation in these two viruses, further emphasizing the high conservation of NS1′s structure and function [36, 37]. The NS1 aa sequences of all PALVs contain only 14 conserved cysteine residues. However, like EHDV and BTV, PALV's NS1 also has two cysteine residues at positions 336 and 339, suggesting that PALV's NS1 may also play a crucial role in protein formation and that strong purifying selection is acting on it. Phylogenetic analyses show that NS1 forms distinct regional topotypes (Asia, Australia, Africa, and CI groups), and the three isolates in this study were clustered in the Asia group. The distinct phylogenetic separation of strains based on geographic origin suggests that regional factors likely influence selection, reflecting variations in cellular replication among various regional groups.

The VP7 protein of the outer core, encoded by Seg-7, serves as the ligand for binding to the cellular receptors of susceptible insect cells. One feature of VP7 is the presence of many synonymous mutations at the third base position, which results in significant differences in percentage identity between the nt and aa sequences (≥84.3% [*μ* = 89.6%] nt and ≥97.7% [*μ* = 99.3%] aa). A highly conserved Arg-Gly-Asp (RGD) motif in VP7 was found in TIBOV, BTV, and EHDV, and it was linked to the virus's attachment to insect cells [38, 39]. This RGD motif is also conserved in all analyzed VP7 segments of PALVs at positions 178–180. This suggests that VP7 faces significant functional constraints that limit the variation of aas. The high degree of conservation also validates the use of diagnostic tests for PALV based on Seg-7 and VP7. The VP7 phylogenetic tree demonstrates similar relationships to those observed in NS1 proteins, with distinct groups based on geographic origin. However, a strain isolated from Japan (ON-14/E/17) and one isolated from Australia (CSIRO 82) were clustered in the Australia and Asia groups, respectively. The results indicate that genome segment reassortment has taken place between different groups. This type of reassortment has already been observed in BTV. For instance, BTV-9 was isolated in Turkey and grouped with other eastern strains of BTV, while Seg-8 clustered with western strains [40]. In India, a strain of BTV-2, classified as an eastern virus, was found to be clustered with western strains of BTV in Seg-2 [40]. The three strains in this study were clustered within a large branch of the Asia group, alongside other strains from Japan, India, and China. They shared 93.0%–99.8% (*μ* = 96.7%) and 98.5%–100% (*μ* = 99.6%) nt/aa sequence identity with them, indicating that the three PALVs share a common ancestor with the Japanese and Indian strains.

Unlike NS1 and VP7, which have many synonymous mutations, VP6 exhibits a somewhat lower identity at the aa level. In the Asia group, the identity in Seg-9/VP6 decreases from ≥93.0% (*μ* = 96.5%) at the nt level to ≥91.9% (*μ* = 96.4%) at the aa level. The Australia group exhibits a similar trend, with identity dropping from 97.9% (*μ* = 98.3%) at the nt level to 97.4% (*μ* = 98.0%) at the aa level. Similarly, in the Africa group, the identity decreases from 95.3% (*μ* = 97.0%) nt to 93.7% (*μ* = 96.3%) aa, while in the CI group, it drops from 93.2% nt to 92.2% aa. If core proteins evolve through accumulated point mutations, these findings suggest that the various VP6 topotypes emerged relatively recently in PALV's evolutionary timeline. However, the C-terminal region (the last 58 aa), conserved among different serotypes with aa sequence identity ≥94.8% (*μ* = 98%), may be structurally and/or functionally important. In addition, Yamakawa et al. [12] reported that alterations in aas may not impact the structure or function of the VP6 protein, which acts as an RNA helicase, and that the protein's hydrophilic properties and secondary structure remain similar across various strains. Anthony et al. [40] analyzed EHDV VP6 and found that the protein experiences less selective pressure, allowing coding sequence variations.

The most variable components of *Orbivirus* are the serotype determinant, the cell attachment protein VP2, and its encoding segment. Seg-2/VP2 is the most variable segment of all PALV genomes, displaying ≥46.8% (*μ* = 60.7%) identity at the nt level and only ≥35.4% (*μ* = 54.4%) at the aa level. This variability indicates the presence of nonsynonymous mutations in the coding sequence of VP2 and suggests that selective pressure is likely favoring specific genetic variants. This result aligns with analyses of BTV VP2, which showed >41.0% identity at the nt level and >27.0% at the aa level [41]. Similarly, the VP2 of EHDV demonstrated >45.8% nt identity and >31.1% aa identity [42]. The significant variability in the VP2 protein of BTV and EHDV is thought to be due to its role as the outermost protein, making it the primary target for immune selective pressure from neutralizing antibodies produced by vertebrate hosts. In the VP2 phylogenetic tree, the three isolates are clustered in Groups A, E, and F and are shown to belong to the Chuzan, Bunyip Creek, and D'Aguilar serotypes, respectively. Group A included three serotypes: Chuzan/Kasba, Vellore, and Abadina, but the nt and aa identity between Vellore and Chuzan/Kasba was ≥88.1%/92.6% (Supporting Information 6: Table S6). Therefore, Vellore may be of the same serotype as Chuzan/Kasba. Abadina showed ≥79.4%/85.0% (nt/aa) identity to the Chuzan/Kasba and Vellore serotypes, and while their serotype relationship may require further study, there is undoubtedly a close genetic relationship between them. The identity between CSIRO Village and Gweru serotypes in Group B showed ≥99.6%/99.5% (nt/aa), indicating that they belong to the same serotype. Similarly, serotype Apies River shared 97.3%/98.1% (nt/aa) identity with Marondera in Group E, so it can also be considered that they belong to the same serotype. Therefore, this study proposes to classify the current PALV serotypes into eight types tentatively: Chuzan/Kasba/Vellore (1), Abadina (2), Gweru/CSIRO Village (3), Marrakai (4), Petevo (5), Bunyip Creek (6), Apies River/Marondera (7), and D'Aguilar (8). However, further studies are needed to make a final determination.

The SNT results indicated that three serotypes of PALV (CHUV, BCV, and DAGV) are present in Yunnan, China, each exhibiting varied prevalence patterns. Overall, the serotype CHUV exhibits the highest positive rate. This result aligns with the isolation of multiple CHUV strains from cattle and yaks in several provinces of China, including Yunnan, Guangxi, and Gansu [16–18]. However, 59 (16.9%) of the 350 cattle serum samples were positive for CHUV in Yunnan, which was significantly higher than the CHUV infection rate in yaks (7%) in Gansu [18] and the infection rate in cattle (3.04%) in the neighboring country, South Korea [43]. Warm, rainy summers in much of Yunnan support large populations of biting insects like mosquitoes and *Culicoides* spp., making this region one of the most active areas in China for insect-borne diseases. In the study, three serotypes of PALV were isolated from *Culicoides* spp. captured in cattle corrals, neutralizing antibodies were detected in cattle serum obtained from the same areas. The findings indicate that *Culicoides* spp. may act as a potential vector for PALV, with cattle potentially serving as a natural reservoir for the disease. However, further research is needed to understand the vector competence of Culicoides for PALV fully. The study also confirmed the widespread presence of PALV infections among local cattle populations, supporting the notion that the virus naturally circulates within these animals. Still, further intensive surveillance and research are necessary to determine the extent to which PALV poses a threat to cattle and other livestock.

## 5. Conclusions

This study details the isolation, serotype, and genomic analysis of three PALVs. Remarkably, this is the first instance of isolating PALV, specifically BCV and DAGV serotypes, from the *Culicoides* spp. vector in China. The results also clarify the genetic relationships between the PALVs identified in China and those found in Japan, Australia, and other regions. This seroepidemiological investigation highlights three serotypes of the PALV (CHUV, BCV, and DAGV) epidemic in Yunnan cattle with varying positive rates and spatial distribution. These outcomes indicate the presence of BAV and DAGV beyond CHUV in China, suggesting that these viruses occur naturally in Southwest China and infect domestic cattle in the region. The results of this study could help improve the diagnosis and prevention of PALV infection in China and facilitate the sharing of beneficial information on surveillance efforts among neighboring countries.

## Figures and Tables

**Table 1:** Isolation details of three PALV strains isolated from Yunnan province, China.

Strain	Serotype	Geographic origin (county)	Longitude and latitude	Altitude (m)	Collection date	GenBank accession number
MY_C17	D'Aguilar	Jinghong	100°50′58″ E and 22°5′18″ N	814	July 7, 2022	PQ536738–PQ536747
ML_C40	Chuzan	Mengla	101°22′23″ E and 21°25′10″ N	569	August 14, 2022	PQ536728–PQ536737
SZ_C06	Bunyip Creek	Shizong	104°17′9″ E and 24°37′38″ N	974	July 16, 2023	PQ536748–PQ536757

**Table 2:** Summary of percentage sequence identities of nucleotide (nt) and amino acid (aa) for NS1, VP7, and VP6 for Asia, Australia, Africa, CI groups, and all strains, respectively.

Varied geographical origins of PALV	Asia group		Australia group		Africa group		CI group		All strains	
	nt	aa	nt	aa	nt	aa	nt	aa	nt	aa
Seg-5/NS1	≥94.3	≥96.8	≥94.9	≥97.7	≥97.4	≥98.1	83.5	93.5	≥80.9	≥91.9
	*μ* = 97.5	*μ* = 98.6	*μ* = 96.1	*μ* = 98.5	*μ* = 98.5	*μ* = 99.5	-	-	*μ* = 89.5	*μ* = 96.5

Seg-7/VP7	≥90.3	≥97.7	≥89.7	≥99.7	≥96.9	≥99.1	89.5	99.4	≥84.3	≥97.7
	*μ* = 95.7	*μ* = 99.5	*μ* = 94.6	*μ* = 99.9	*μ* = 98.4	*μ* = 99.8	-	-	*μ* = 89.6	*μ* = 99.3

Seg-9/VP6	≥93.0	≥91.9	≥97.9	≥97.4	≥95.3	≥93.7	93.2	92.2	≥86.4	≥84.5
	*μ* = 96.5	*μ* = 96.4	*μ* = 98.3	*μ* = 98.0	*μ* = 97.0	*μ* = 96.3	-	-	*μ* = 91.7	*μ* = 91.0

*Note: μ* is the mean, “-” is not available.

**Table 3:** Summary of percentage sequence identities of nucleotides (nt) and amino acids (aa) between different groups and within each group.

Different evolutionary branches of PALV	Group A	Group B	Group C	Group D	Group E	Group F
Group A	≥79.4/85.0	≤53.1	≤43.9	≤44.6	≤41.6	≤40.6
Group B	≤58.0	≥99.4/99.4	≤45.0	≤44.4	≤38.9	≤37.5
Group C	≤53.1	≤53.9	-	52.3	≤39.6	≤38.4
Group D	≤52.2	≤52.2	58.1	-	≤39.4	≤36.9
Group E	≤51.8	≤49.8	≤50.5	≤49.1	≥70.9/73.9	≤48.6
Group F	≤49.8	≤49.6	≤49.4	≤48.0	≤54.9	≥86.9/88.5

*Note:* Below the diagonal is nucleotide identity and above the diagonal is amino acid identity. “-” is not available.

**Table 4:** The detection of CHUV, BCV, and DAGV neutralizing antibodies in cattle in three counties of Yunnan province.

Location	Sample numbers	Numbers of positive sample (positive rate [%])			
		BCV	DAGV	CHUV	PALV
Jinghong	112	3 (2.7)	9 (8.0)	20 (17.9)	32 (28.6)
Mengla	106	3 (2.8)	8 (7.5)	18 (17.0)	29 (27.4)
Shizong	132	6 (4.5)	7 (5.3)	21 (15.9)	34 (25.8)
Total	350	12 (3.4)	24 (6.9)	59 (16.9)	95 (27.1)

## Data Availability

The nucleotide sequences of the complete genome sequences of ML_C40, MY_C17, and SZ_C06 strains obtained in this study were submitted to the GenBank database under accession numbers PQ536728–PQ536737, PQ536738–PQ536747, and PQ536748–PQ536757, respectively.

## Supporting Information

Supporting Information 1 Table S1: The background information and GenBank accession numbers of all virus strains used in this study.

Supporting Information 2 Table S2: Lengths of dsRNA segments 1–10, encoded putative proteins, 5′ and 3′ NCRs of the three PALV strains.

Supporting Information 3 Table S3: Matrix of NS1 pairwise identity percentages of nucleotide (lower left) and amino acid (upper right) between different strains of PALV.

Supporting Information 4 Table S4: Matrix of VP7 pairwise identity percentages of nucleotide (lower left) and amino acid (upper right) between different strains of PALV.

Supporting Information 5 Table S5: Matrix of VP6 pairwise identity percentages of nucleotide (lower left) and amino acid (upper right) between different strains of PALV.

Supporting Information 6 Table S6: Matrix of VP2 pairwise identity percentages of nucleotide (lower left) and amino acid (upper right) between different strains of PALV.
