# Supplementary material for: Isolation and Genetic Characterization of Three Palyam Serogroup Orbiviruses From Culicoides Spp. and Associated Infections in Cattle in Yunnan, China
Source: Transbound Emerg Dis. 2025 Aug 25;2025:6615175. doi: 10.1155/tbed/6615175 (PMC12401601; doi:10.1155/tbed/6615175)
Supplement: Supporting Information 2 — Table S2: Lengths of dsRNA segments 1–10, encoded putative proteins, 5′ and 3′ NCRs of the three PALV strains. [file 6615175.f2.docx]

Table S2 Lengths of dsRNA segments 1–10, encoded putative proteins, 5' and 3′ NCRs of the three PALV strains.

| Strains | Segment | Protein encoded | Segment length (bp) | Size of protein (aa) | Protein molecular mass (kDa) | G+C content (%) | 5' NCR (bp) | Terminal sequence (5'–3') | 3' NCR (bp) | Stop-codon | GenBank Accession no. |
| --- | --- | --- | --- | --- | --- | --- | --- | --- | --- | --- | --- |
| ML_C40 | Seg-1 | VP1 (Pol) | 3930 | 1295 | 149.22 | 38.52 | 22 | **GUUAAA**U---A**CUUAC** | 20 | UAG | PQ536728 |
|  | Seg-2 | VP2 (OC1) | 3055 | 1002 | 117.49 | 38.07 | 14 | **GUUAAA**A---G**CUUAC** | 33 | UAG | PQ536729 |
|  | Seg-3 | VP3 (T2) | 2774 | 904 | 103.86 | 39.51 | 13 | **GUUAAA**U---A**CUUAC** | 46 | UAG | PQ536730 |
|  | Seg-4 | VP4 (Cap) | 1967 | 640 | 74.60 | 38.08 | 9 | **GUUAAA**A---A**CUUAC** | 35 | UAG | PQ536731 |
|  | Seg-5 | NS1 (Tup) | 1764 | 545 | 64.14 | 37.47 | 35 | **GUUAAA**A---A**CUUAC** | 91 | UAA | PQ536732 |
|  | Seg-6 | VP5 (OC2) | 1610 | 521 | 59.05 | 40.43 | 20 | **GUUAAA**A---G**CUUAC** | 24 | UAG | PQ536733 |
|  | Seg-7 | VP7 (T13) | 1151 | 348 | 37.98 | 44.57 | 18 | **GUUAAA**A---G**CUUAC** | 86 | UGA | PQ536734 |
|  | Seg-8 | NS2 (Vip) | 1058 | 333 | 37.13 | 40.55 | 20 | **GUUAAA**A---A**CUUAC** | 36 | UAG | PQ536735 |
|  | Seg-9 | VP6 (Hel) | 877 | 272 | 29.71 | 41.51 | 19 | **GUUAAA**A---A**C**A**UAC** | 39 | UAG | PQ536736 |
|  | Seg-10 | NS3 (VRP) | 728 | 211 | 23.61 | 40.66 | 18 | **GUUAAA**A---A**CUUAC** | 74 | UAA | PQ536737 |
|  | Total |  | 18914 | 6071 | 696.79 | 39.31 | 188 | **GUUAAA**-------**C^U^/_A_UAC** | 484 |  |  |
| MY_C17 | Seg-1 | VP1 (Pol) | 3930 | 1295 | 149.34 | 37.81 | 22 | **GUUAAA**U---A**CUUAC** | 20 | UAG | PQ536738 |
|  | Seg-2 | VP2 (OC1) | 3022 | 992 | 116.13 | 37.46 | 16 | **GUUAAA**U---A**CUUAC** | 17 | UGA | PQ536739 |
|  | Seg-3 | VP3 (T2) | 2774 | 904 | 103.88 | 39.58 | 13 | **GUUAAA**U---A**CUUAC** | 46 | UAG | PQ536740 |
|  | Seg-4 | VP4 (Cap) | 1967 | 640 | 74.65 | 37.06 | 9 | **GUUAAA**A---A**CUUAC** | 35 | UAG | PQ536741 |
|  | Seg-5 | NS1 (Tup) | 1764 | 545 | 64.14 | 37.81 | 35 | **GUUAAA**A---A**CUUAC** | 91 | UGA | PQ536742 |
|  | Seg-6 | VP5 (OC2) | 1610 | 521 | 58.88 | 39.19 | 20 | **GUUAAA**U---A**CUUAC** | 24 | UAG | PQ536743 |
|  | Seg-7 | VP7 (T13) | 1151 | 348 | 37.99 | 44.22 | 18 | **GUAAAA**A---G**CUUAC** | 86 | UGA | PQ536744 |
|  | Seg-8 | NS2 (Vip) | 1058 | 333 | 37.27 | 40.55 | 20 | **GUAAAA**A---A**CUUAC** | 36 | UAG | PQ536745 |
|  | Seg-9 | VP6 (Hel) | 877 | 272 | 29.67 | 40.71 | 19 | **GUUAAA**A---A**C**A**UAC** | 39 | UAG | PQ536746 |
|  | Seg-10 | NS3 (VRP) | 728 | 211 | 23.57 | 40.8 | 18 | **GUUAAA**A---A**CUUAC** | 74 | UAA | PQ536747 |
|  | Total |  | 18881 | 6061 | 695.52 | 38.85 | 190 | **GUUAAA**-------**C^U^/_A_UAC** | 468 |  |  |
| Strains | Segment | Protein encoded | Segment length (bp) | Size of protein (aa) | Protein molecular mass (kDa) | G+C content (%) | 5' NCR (bp) | Terminal sequence (5'–3') | 3' NCR (bp) | Stop-codon | GenBank Accession no. |
| SZ_C06 | Seg-1 | VP1 (Pol) | 3930 | 1295 | 149.34 | 37.86 | 22 | **GUUAAA**U---A**CUUAC** | 20 | UAG | PQ536748 |
|  | Seg-2 | VP2 (OC1) | 3064 | 1006 | 117.18 | 39.46 | 16 | **GUUAAA**U---A**CUUAC** | 27 | UGA | PQ536749 |
|  | Seg-3 | VP3 (T2) | 2774 | 904 | 103.84 | 39.33 | 13 | **GUUAAA**U---A**CUUAC** | 46 | UAG | PQ536750 |
|  | Seg-4 | VP4 (Cap) | 1967 | 640 | 74.67 | 37.98 | 9 | **GUUAAA**A---A**CUUAC** | 35 | UAG | PQ536751 |
|  | Seg-5 | NS1 (Tup) | 1764 | 545 | 64.13 | 37.47 | 35 | **GUUAAA**A---A**CUUAC** | 91 | UGA | PQ536752 |
|  | Seg-6 | VP5 (OC2) | 1610 | 521 | 58.97 | 40.93 | 19 | **GUUAAA**A---A**CUUAC** | 22 | UAG | PQ536753 |
|  | Seg-7 | VP7 (T13) | 1151 | 348 | 37.99 | 43.79 | 18 | **GUAAAA**A---A**CUUAC** | 86 | UGA | PQ536754 |
|  | Seg-8 | NS2 (Vip) | 1058 | 333 | 37.27 | 40.26 | 20 | **GUAAAA**A---A**CUUAC** | 36 | UAG | PQ536755 |
|  | Seg-9 | VP6 (Hel) | 877 | 272 | 29.67 | 41.28 | 19 | **GUUAAA**A---A**C**A**UAC** | 39 | UAG | PQ536756 |
|  | Seg-10 | NS3 (VRP) | 728 | 211 | 23.60 | 39.97 | 18 | **GUUAAA**A---A**CUUAC** | 74 | UAA | PQ536757 |
|  | Total |  | 18923 | 6075 | 696.66 | 39.31 | 189 | **GUUAAA**-------**C^U^/_A_UAC** | 476 |  |  |

Conserved nucleotide sequences in 5′- and 3′-terminals are shown in bold. RNA-dependent RNA Polymerase (Pol), Outer capsid protein (OC1), Major subcore protein (T2), Minor core protein-Capping enzyme (CaP), Tubule protein (TuP), Outer capsid protein (OC2), Major core-surface protein (T13), Viral inclusion body protein (ViP), Minor core protein-helicase enzyme (Hel), Virus release protein (VRP).
